# Early evidence from South Carolina’s Medicare-Medicaid dual-eligible financial alignment initiative: an observational study to understand who enrolled, and whether the program improved health?

**DOI:** 10.1186/s12913-018-3721-6

**Published:** 2018-11-29

**Authors:** Brian K. Chen, Y. Tony Yang, Rachelle Gajadhar

**Affiliations:** 1 University of South Carolina, 915 Greene Street Suite 354, Columbia, SC 29208 USA; 2 George Washington University, 1919 Pennsylvania Ave. NW Suite 500, Washington, DC, 20006 USA; 3 University of South Carolina, 3010 Farrow Rd. Suite 300, Columbia, SC 29203 USA

**Keywords:** Medicare, Medicaid, Payment systems; aging/elderly/geriatrics

## Abstract

**Background:**

Individuals dually eligible for Medicare and Medicaid coverage are among the sickest patients in the United States. Prior literature has identified a lack of care coordination or even conflicts of interest between the two programs as barriers to more efficient care and better health outcomes among dual-eligibles. The purpose of this study is to assess characteristics of dual eligibles who participated in South Carolina’s 2015 voluntary Medicare-Medicaid financial alignment demonstration project, and to evaluate whether their participation led to better observable health outcomes.

**Methods:**

We obtained all inpatient and emergency department visits, and all Medicaid outpatient visits of individuals identified as Medicare-Medicaid dual eligibles from 2011 to 2016 from South Carolina’s Revenue and Fiscal Affairs Office. We employed logistic regressions to assess the characteristics of participants and quitters in the Medicare-Medicaid financial alignment demonstration project. To evaluate the impact of participation on health outcomes, we used an event study analysis that examines trends in outcomes over time, with participation in the demonstration project as the triggering event, and a difference-in-differences methodology that compares changes in health outcomes before and after participation in the demonstration project compared with a control group.

**Results:**

Urban patients, female patients, and patients with heart problems, social and mental disorders, and importantly, patients with multiple comorbidities (as indicated by a higher Charlson comorbidity index) are less likely to join South Carolina’s demonstration project. Once having joined, female patients and patients with a higher Charlson index appear to be more likely to quit. Those who joined did not appear to enjoy better health outcomes in the short time frame.

**Conclusions:**

Policy makers should explore and address reasons why dual eligibles with complex health problems hesitate to join the alignment project, and continue to monitor whether such a program improves health given that a prolonged period of exposure to the program may be required to achieve better health among the nation’s most vulnerable patients.

## Background

Health insurance coverage in the United States (US) is fragmented and consists of a mixture of public and private sources of funding. The two major sources of public health insurance in the US are Medicare, which primarily covers adults aged 65 and older,^1^ and Medicaid, which primarily covers low-income individuals and families.^2^ In 2017, these two programs collectively provided insurance coverage to approximately 37% of the US population [1]. While most individuals with public health insurance qualify for either Medicare or Medicaid, some qualify for both types of coverage because of old age and low income.^3^ These individual, consisting primarily of the elderly poor and known as dual eligibles, have long been the subject of extensive policy debate because of their poor health, complex health conditions and attendant high costs of care [2, 3].

Dual eligibles are more likely than non-dual Medicare beneficiaries to report poor health (19% versus 7%), and are more likely to have limitations in activities of daily living (59% versus 33%) [4]. They are more than twice as likely as non-dual Medicare beneficiaries to have at least three chronic conditions, and three times as likely to have been diagnosed with a mental illness [5]. The 11.7 million Americans dually enrolled for Medicare and Medicaid benefits account for a disproportionately large share of expenditures in both Medicare and Medicaid. In 2012, dual eligibles represented 20% of Medicare enrollees but 34% of the federal program’s total expenditures [6]. They accounted for only 15% of Medicaid enrollees but over 33% of Medicaid spending [6]. In the same year, the combined federal and state expenditures to care for dual eligibles exceeded $306 billion [6].

These figures underlie the policy significance of testing appropriate models of care and payment for this particularly vulnerable group of elderly Americans. Because part of their care is covered by Medicare and part is covered by Medicaid, dual eligibles have to navigate both programs to receive care for their medical conditions. For dual eligibles, the lack of *clinical integration* between two separate programs has been faulted for lower quality of care [7, 8]. Separate delivery systems for dual-eligibles generally require them to seek medical care from poorly coordinated providers that have no incentive to interact given that they are paid by different insurers [9, 10]. The lack of interaction between providers, compounded by differential practices and poor communication, creates significant barriers to receiving coordinated care that is required for dually eligible individuals with multiple and complex chronic illnesses.

Moreover, the lack of *financial integration* of payments between (and at times, within) Medicare and Medicaid has resulted in conflicting incentives and cost shifting between payers. This lack of alignment in financial incentives between Medicare and Medicaid has been faulted to lead to higher overall healthcare costs [11]. Dual eligibles who are discharged from acute inpatient care often find their discharge delayed because appropriate long-term care in a nursing home or in the patient’s home cannot be arranged efficiently. The lack of a single payer exacerbates this problem because Medicaid, which pays for the long-term care, has no incentive to reduce acute hospitalization costs covered by Medicare [9]. Moreover, Medicaid provides less extensive coverage for home- and community-based programs than long-term care, creating an incentive for dually eligible individuals to seek costly institutional care even their preference is to remain in lower-cost community settings [9].

The literature on the consequences of care fragmentation among dual eligibles is vast. It is estimated that Medicare spent $3 billion in 2005 on potentially avoidable hospitalizations for dually eligible individuals [12]. Among the dual eligibles in long-term care or skilled nursing facilities covered primarily by Medicaid, more than two-thirds were hospitalized at least once, and 39% of these hospitalizations were deemed avoidable because they could have been prevented with timely and appropriate treatment at lower levels of care [13]. Overall, the literature suggests that rates of hospitalizations for some ambulatory care sensitive conditions among dual eligibles are twice those of the rest of the Medicare population [14].

As part of the Affordable Care Act, the Center for Medicare/Medicaid Services (CMS) encouraged state Medicare-Medicaid Financial Alignment Initiative demonstration projects to address conflicts in program incentives and access to care [15]. These demonstration projects are called financial alignment initiatives because they seek to align the conflicting financial incentives that arise when Medicare and Medicaid pay for different aspects of a patient’s health care. Fundamentally, financial conflicts of interest exist because the actions of one payer has financial implications for the other payer that also covers the same individuals. But the first payer only has the incentive to assess implications for its own costs, rather than the costs borne by the second payer. As a result, the first payer may make decisions that result in modest cost savings to itself with significantly higher cost implications for the second payer [10, 16].

Previous literature suggested that coordination of care delivery could be effective in improving clinical outcomes for patients with chronic illnesses [17–19]. The CMS Financial Alignment Initiative builds upon such integrated *clinical care* models by *financially* integrating Medicare and Medicaid for dual-eligible beneficiaries. The financial integration of the two payers is theorized to overcome the inherent agency problems with multiple payers cover different aspects of a single population’s health [20]. Thirteen states, including South Carolina (the subject of our study), submitted memoranda of understanding to CMS that were approved [21, 22]. Each demonstration state designed and implemented its own version of the Financial Alignment project, and in South Carolina, the particular version of the demonstration project is known as Healthy Connections Prime (HCP). In this state, Medicare and Medicaid combined their payments to “Coordinated and Integrated Care Organizations” (CICOs), which would coordinate and integrate the care of dual eligibles enrolled in HCP. Implemented in phases with a voluntary opt-out option, HCP is based on the patient-centered medical home model with increased attention to primary and preventive care; adoption of best practices in care coordination and holistic team-based care; establishment of health information technology to provide efficient care; and integrated payment structures to address conflicting incentives [22].

To South Carolinians, HCP was promoted as an integrated program superior to separate coverage under Medicare and Medicaid. On the HCP website, the program is billed as “one card, one plan” and described to be “an enhanced program that combines all of the benefits of Medicare and […] Medicaid under a single Medicare-Medicaid Plan to make it easier to get the health services you need [23].” The primary goals of HCP are listed as:

- Better Care: by making it easier to get all of your Medicare, Medicaid and Medicare Part D services from a single health plan
- Better Value: through a care team and a care manager that works directly with you or your loved one and providers to make sure you or your loved one get needed health services
- Better Health: through flexible benefits that help you stay at home with family as long as possible [23]

By design, then, South Carolina’s version of the Financial Alignment Initiative, or HCP, is meant to overcome the fragmented care faced by dual eligibles by implementing both *clinical* and *financial* integration of Medicare and Medicaid. The question, then, is whether such voluntary opt-out integrated clinical care and financial incentives programs attract enrollment, and if so, do they improve health outcomes? Thus far, there exists only a small body of literature on the utilization, health and financial outcomes of such programs, warranting further empirical study [7]. A study by Grabowski of eight demonstration programs (excluding South Carolina’s project because of the recency of its implementation), found that only 26.7% of qualified dual-eligibles actually passively enrolled, and the programs experienced high opt-out rates, casting doubt on the effectiveness of passive enrollment mechanisms [24]. An analysis of Massachusetts’ Senior Care Options (SCO) for dual eligibles found no statistically significant effect of SCO on hospital readmission rates, suggesting that managing care for dual eligibles in this manner may not be sufficient to significantly improve their certain aspects of their health status [25].

Our study proposes to contribute to the literature by analyzing the CMS Demonstration Project as implemented in South Carolina. We aim to answer two empirical questions: (1) Who enrolled in HCP, given its voluntary opt-out nature, and (2) whether enrollment in HCP reduced avoidable ED encounters, inpatient admissions, and discharge to skilled nursing facilities. Our study adds to the literature in two important ways. First, it complements the previous Grabowski et al. study by investigating a demonstration state explicitly excluded from their study. Second, this research studies a vulnerable elderly population in a state with particularly high chronic illness burden and health disparities [26]. Finally, by understanding the characteristics of those who opted not to enroll, and those who disenrolled after enrolling, we gain a better understanding of the type of patients that HCP is failing to reach. This will shed important light on possible failures and barriers in the HCP program if lack of participation is associated with specific patient characteristics, such as having more complex medical conditions. It is hoped that our study, in combination with other evaluations of the CMS Financial Alignment Initiative demonstration states, will help policymakers further refine a program meant to improve the health of and decrease costs associated with a particularly vulnerable elderly population.

## Methods

### Data

Our data consist of all-payer claims data for all inpatient and emergency department (ED) encounters for Medicare-Medicaid dually eligible enrollees in South Carolina from January 2011 to December 2016. Additionally, we obtained data on all outpatient encounters during the same period for which Medicaid is the primary payer. We obtain all such data from South Carolina’s Revenue and Fiscal Affairs Office (RFA). We derive information on patient demographics from the dual eligible enrollee’s Medicaid master beneficiary file, which also includes enrollees’ month-by-month Medicare/Medicaid as well as HCP enrollment status.

### Statistical analysis

#### Dependent variables

All dependent variables (charges for avoidable ED/hospitalization, inpatient lengths of stay (LOS), and skill nursing facility (SNF) placement) are taken directly from the all-payer ED and inpatient data and aggregated by patient at the quarter level. An avoidable ED visit is defined as a principal diagnosis with 90% of being “emergency care need – preventable” according to the New York University ED Algorithm [27]. An inpatient admission is considered avoidable if its principal diagnosis is one of the Prevention Quality Indicators compiled by the Agency for Healthcare Research and Quality [28].

#### Independent variables

Demographic information on patients (age, female sex, ethnicity dummy variables “black” and “other race”) and Healthy Connections Prime (HCP) enrollment status are derived from the Medicaid master beneficiary file. Each patient’s baseline Charlson comorbidity index (using the Deyo method) [29] and presence/absence of all Elixhauser comorbidities [30] were calculated using all diagnosis codes in the Medicaid outpatient, and all-payer ED and inpatient encounters for the entire year of 2014 or 2015, depending on the empirical specification.

#### Factors associated with joining HCP

We investigate factors associated with joining South Carolina’s HCP and leaving HCP using logistic regressions with the unit of observation as the individual dually eligible enrollee. For the analysis on joiners, we identified all dual eligibles in 2015 and 2016, and all individuals who ever enrolled in HCP, even for a single month. We regressed whether an individual enrolled in HCP on age, gender (female), race/ethnicity (black, other race, with white race as the reference), Charlson comorbidity index in the previous year (2014 or 2015) and a vector of dummy variables for all Elixhauser comorbidities.

#### Factors associated with quitting HCP

Here, we limit our cohort to all individuals who ever joined HCP. Then, from this cohort, we define those who quit HCP as individuals who remain dually enrolled for at least 2 months after quitting HCP (to exclude “quitters” who did so through loss of dual eligibility). We repeated the logistic regression as above with this substantially smaller cohort, with HCP quitter status as the dependent variable.

All logistic regression results are presented as odds ratios.

#### Health outcomes following HCP enrollment

To study the impact of enrolling in HCP on key measures of health outcomes, we perform two separate analyses – an event study that takes into account the different HCP enrollment dates of dual eligibles, adjusted for race, gender, Charlson comorbidity index, and Elixhauser comorbidities at baseline; and a difference-in-differences (DID) analysis of differences in key outcomes between “ever HCP” and “Never HCP” dual eligibles.

We define “Ever HCP” enrollers as those who enrolled and remained in HCP for 6 months or more. We discard data associated with individuals who enrolled in HCP but quit within 6 months. The remaining dual eligibles were assigned to the control group, or the “Never HCP” group.

##### Event study

Because HCP enrollers joined the program at different times, we conduct a regression-adjusted event study analysis. Here, we normalize time (in quarters) before and after an individual joined HCP. For example, a person who joined HCP in 2015 quarter 1 has the quarter variable Q_0_ set as 1. For this person, Q_1_ is 1 for 2015 quarter 2, and Q_2_ is 1 for 2015 quarter 3. Likewise, Q_− 1_ is 1 for 2014 quarter 4, Q_− 2_ is 1 for 2014 quarter 3, Q_− 3_ is 1 for 2014 quarter 2, and so on. For never HCP patients, we randomly assigned them to one of eight possible enrollment start quarters to serve as control. We limit our analysis to seven quarter before and after each person’s HCP enrollment quarter, using the following specification:

$$ {y}_{iq}={\alpha}_0+\sum \limits_{q=-7}^7{\beta}_q\cdot {Q}_{iq}\cdot {treatment}_i+\gamma \cdot {X}_i+{\varepsilon}_{iq} $$

In the specification above, *y* represents the various outcomes of interest. *Q*_*iq*_ is the normalized quarter for each individual, *treatment* is 1 if the patient has ever joined HCP for at least 6 months, and 0 otherwise. *Q*_*i0*_ is excluded from the regression above so that all other quarters are relative to Q_i0_, or the quarter individual *i* joins HCP. ***X*** represents a vector of demographic and clinical independent variables described above, and ε represents the random error. The coefficients β_− 7_, β_− 6_, … β_− 1_, β_1_, β_2_, … β_7_ on the 14 *Q*_*iq*_ *× treatment* variables represent the average differences in outcomes between the Ever HCP patients and Never HCP patients in each of those quarters. In other words, a 0 coefficient means that there is no difference in outcome between the two groups in that quarter. A negative coefficient on *Q*_*7*_ signifies that the treatment group (Ever HCP) has fewer numbers of the outcome, e.g., avoidable ED visits, in the seventh quarter after HCP enrollment, and vice versa for a positive coefficient.

##### Difference-in-differences

Our second approach consisted of a DID analysis of key outcomes for Ever and Never HCP dual eligibles. The specification used here is:

$$ {y}_{iq}={\alpha}_0+{\beta}_1\cdot {post}_{iq}+{\beta}_2\cdot {treatment}_i+{\beta}_3\cdot {post}_{iq}\times {treatment}_i+\gamma \cdot {X}_i+{c}_i+{\varepsilon}_{iq} $$

Here, *post* is 1 for observations in the post-enrollment period (including the artificial post-period for Never HCP enrollees), and 0 otherwise. *Treatment* is 1 for Ever HCP patients, and 0 for Never HCP patients. *Post×treatment* is an interaction term for these two terms, *c*_*i*_ represents a vector of county fixed effects, and *y* and ***X*** are as defined in the event study analysis. The DID regressions are in effect a less refined analysis than the event study, aggregating all pre- and post- quarters to estimate β_3_. This key coefficient represents the difference between the post and pre-periods of the Ever HCP group minus the difference between the post- and pre-periods of the Never HCP group. The event study, on the other hand, shows these differences on a quarter-by-quarter basis.

## Results

**Fig. 1:**
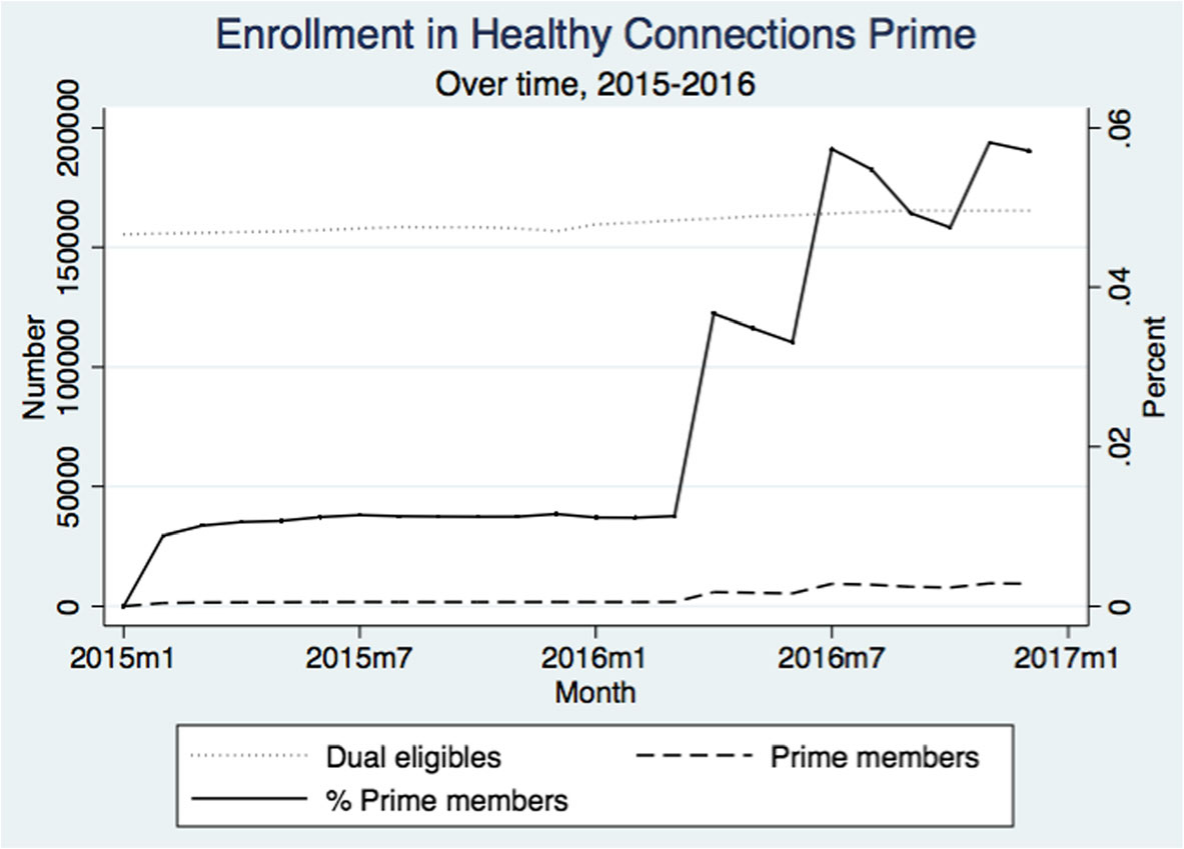
Healthy connections prime enrollment over time

Our data show that of the 260,325 dual eligibles in South Carolina, only 13,370 individuals ever joined HCP. Initial uptake of HCP in the first quarter of 2015, the first enrollment quarter, was relatively modest, with only approximately 4% of eligible individuals joining HCP. The percentage never surpassed 5.7% by the end of the study period in December 2016, when 9442 individuals out of 165,277 dually eligible beneficiaries enrolled in HCP. See Fig. 1.

### Joining or quitting HCP

#### Overall summary

See Table 1. Older, male, and minority (other than black) beneficiaries are more likely to join HCP. Patients with several types of individual comorbidities (hypertension and vascular disease, COPD, diabetes, renal failure, liver disease, AIDS and cancer) are more likely to enroll in HCP. African Americans are more likely to join HCP in specifications not adjusting for comorbidities at baseline, but this association disappears once comorbidities are included. On the other hand, it appears that urban patients, female patients, and patients with heart problems, social and mental disorders, and importantly, patients with multiple comorbidities (as indicated by a higher Charlson comorbidity index) are less likely to join. Once having joined, female patients and patients with a higher Charlson index (in Table 1 specification (5)) appear to be more likely to quit.

**Table 1:** Decision to join or quit prime

Variables	Joiner			Quitter		
	(1) Joined Prime	(2) Joined Prime	(3) Joined Prime	(4) Quit Prime	(5) Quit Prime	(6) Quit Prime
Age	1.060*** (0.000740)	1.060*** (0.000763)	1.061*** (0.000851)	1.000 (0.00249)	0.999 (0.00256)	1.000 (0.00269)
Female	0.984 (0.0197)	0.972 (0.0207)	0.963* (0.0212)	1.240*** (0.0562)	1.223*** (0.0575)	1.149*** (0.0560)
Urban	0.733*** (0.0139)	0.729*** (0.0145)	0.748*** (0.0150)	0.859*** (0.0356)	0.868*** (0.0369)	0.875*** (0.0376)
Black	1.109*** (0.0220)	1.083*** (0.0224)	0.997 (0.0216)	0.846*** (0.0368)	0.837*** (0.0373)	0.864*** (0.0402)
Other race	1.503*** (0.0413)	1.440*** (0.0427)	1.352*** (0.0406)	0.666*** (0.0424)	0.715*** (0.0475)	0.736*** (0.0494)
CHARLSON INDEX		0.938*** (0.00446)	0.861*** (0.0153)		1.060*** (0.0107)	0.940 (0.0366)
Congestive Heart Failure			0.874*** (0.0330)			1.028 (0.0837)
Cardiac Arrhythmias			0.904*** (0.0253)			1.029 (0.0612)
Valvular Disease			1.431*** (0.0489)			1.146* (0.0806)
Pulmonary Circulation Disorders			0.792*** (0.0460)			1.056 (0.128)
Peripheral Vascular Disorders			1.311*** (0.0515)			1.266*** (0.104)
Hypertension, Uncomplicated			1.422*** (0.0327)			1.006 (0.0508)
Paralysis			0.985 (0.0875)			1.233 (0.242)
Other Neurological Disorders			0.726*** (0.0273)			1.071 (0.0892)
Chronic Pulmonary Disease			1.309*** (0.0388)			1.239*** (0.0798)
Diabetes, Uncomplicated			1.207*** (0.0342)			1.322*** (0.0807)
Diabetes, Complicated			1.196*** (0.0538)			1.226** (0.121)
Hypothyroidism			0.977 (0.0304)			1.099 (0.0714)
Renal Failure			1.121** (0.0602)			1.395*** (0.152)
Liver Disease			1.331*** (0.0777)			1.203 (0.153)
Peptic Ulcer Disease Excluding Bleeding			1.468*** (0.132)			1.400* (0.257)
AIDS/HIV			2.128*** (0.362)			1.198 (0.481)
Lymphoma			1.259* (0.163)			1.215 (0.339)
Metastatic Cancer			1.391** (0.204)			1.772* (0.558)
Solid Tumor Without Metastasis			1.786*** (0.0915)			1.066 (0.120)
Rheumatoid Arthritis/Collagen Vascular			1.115** (0.0577)			1.401*** (0.149)
Coagulopathy			0.811*** (0.0543)			0.976 (0.143)
Obesity			0.837*** (0.0343)			0.807** (0.0747)
Weight Loss			0.812*** (0.0351)			0.858 (0.0827)
Fluid and Electrolyte Disorders			0.699*** (0.0202)			0.917 (0.0579)
Blood Loss Anemia			0.914 (0.0996)			0.896 (0.216)
Deficiency Anemia			0.874*** (0.0407)			1.018 (0.102)
Alcohol Abuse			1.046 (0.0711)			0.645** (0.114)
Drug Abuse			0.755*** (0.0591)			1.099 (0.192)
Psychoses			0.900** (0.0419)			0.928 (0.0980)
Depression			(0.0248)			1.143* (0.0805)
Hypertension, Complicated			0.934 (0.0415)			0.882 (0.0776)
Constant	0.00208*** (0.000107)	0.00250*** (0.000133)	0.00211*** (0.000126)	0.333*** (0.0585)	0.333*** (0.0608)	0.303*** (0.0584)
Observations	198,597	170,655	170,655	13,730	12,614	12,614

Robust standard errors in parentheses

*** *p* < 0.01, ** *p* < 0.05, * *p* < 0.1

### Key health outcomes after enrolling in HCP

**Fig. 2:**
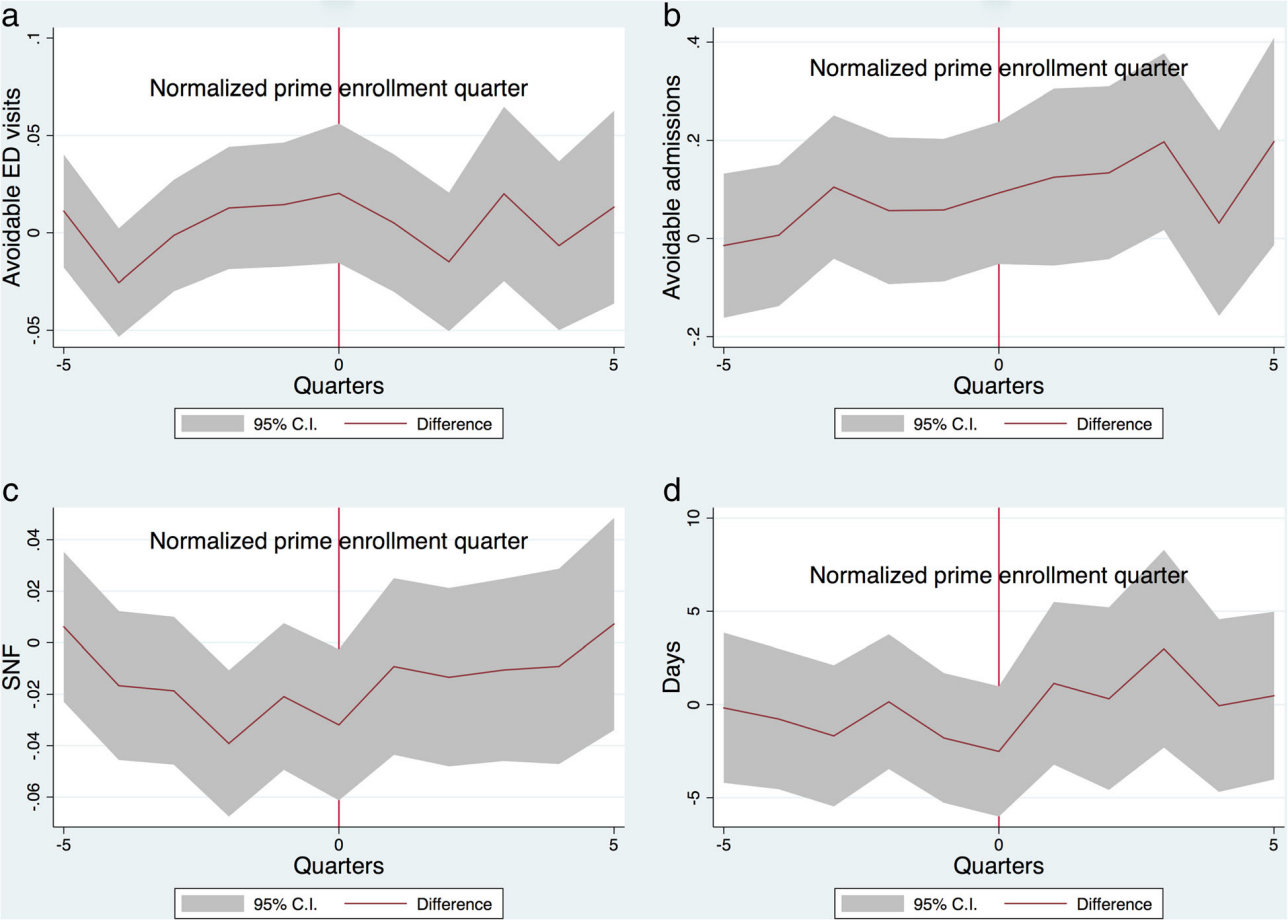
Event studies. The event study figures show the difference in the outcomes (avoidable ED utilization, avoidable inpatient admission, nursing home placement, and inpatient length of stay) between HCP enrollees and non-enrollees five quarters before and after the normalized HCP enrollment date. A difference of 0 means that there are no differences between the two groups. A negative difference means that HCP enrollees have lower instances of the outcome of interest relative to non-enrollees. The 95% confidence interval is represented by the gray area around the difference line. **a** Difference in avoidable ED visits by quarter **b** Difference in avoidable inpatient admissions **c** Difference in SNF placements **d** Difference in inpatient LOS

Overall, the event study analyses and the DID regression agree: Approximately 2 years after HCP first became available, Ever HCP enrollees do not appear to have better indicators of health in terms of avoidable ED visits (Fig. 2a), avoidable inpatient admissions (Fig. 2b), skilled nursing facility placement (Fig. 2c) and LOS (Fig. 2d). The event study analyses also suggest, as in the regressions predicting HCP enrollment or disenrollment, that “healthier” patients (those with lower Charlson index scores) are more likely to join HCP, as the figures show that their utilization for inpatient care in particular was already lower than Never HCP patients before the first available HCP quarter (See Fig. 2b and d).

Finally, in the DID estimation, the coefficients on *post×treatment* are all statistically insignificant, except for those in the specifications for avoidable inpatient admissions and inpatient LOS, which are in fact positive (rather than negative) and significant. These results do not suggest that joining HCP led to a decrease in such clinical outcomes. See Tables 2 and 3.

**Table 2:** Difference-in-differences regressions for ED visits

Variables	Avoidable ED visits		ED charges	
	(1)	(2)	(3)	(4)
Post	0.00844*** (0.000890)	0.00837*** (0.000889)	322.1*** (10.17)	316.7*** (9.999)
Treatment	−0.00618*** (0.00196)	− 0.00699*** (0.00199)	−10.63 (17.65)	−5.899 (17.40)
Post X treatment	0.00673 (0.00465)	0.00671 (0.00466)	16.63 (39.78)	56.39 (38.40)
Age	−6.38e-05** (2.62e-05)	−7.75e-05*** (2.58e-05)	−12.76*** (0.378)	−13.07*** (0.379)
Female	− 0.00627*** (0.000892)	− 0.00625*** (0.000903)	−21.70** (9.524)	−27.76*** (9.408)
Urban	−0.000924 (0.000774)	0.00255* (0.00147)	246.8*** (8.714)	98.32*** (20.29)
Black	0.0138*** (0.000809)	0.0113*** (0.000818)	107.8*** (9.902)	39.95*** (9.582)
Other race	0.0163*** (0.00192)	0.0147*** (0.00183)	103.7*** (13.67)	50.69*** (13.61)
CHARLSON INDEX	−0.00210*** (0.000705)	− 0.00215*** (0.000708)	102.5*** (7.442)	91.49*** (7.341)
Congestive Heart Failure	0.0283*** (0.00175)	0.0282*** (0.00175)	−50.31*** (17.13)	−20.03 (16.75)
Cardiac Arrhythmias	0.0120*** (0.00147)	0.0118*** (0.00143)	421.6*** (14.81)	410.3*** (14.50)
Valvular Disease	0.00437*** (0.00156)	0.00450*** (0.00155)	97.62*** (19.80)	78.02*** (19.34)
Pulmonary Circulation Disorders	−0.00793*** (0.00235)	−0.00827*** (0.00236)	338.5*** (36.36)	331.8*** (35.92)
Peripheral Vascular Disorders	−0.00373** (0.00173)	−0.00360** (0.00175)	−109.5*** (18.44)	−70.24*** (18.12)
Hypertension, Uncomplicated	−0.00550*** (0.000833)	−0.00543*** (0.000854)	81.86*** (9.713)	67.28*** (9.541)
Paralysis	−0.00320 (0.00252)	− 0.00349 (0.00253)	− 217.1*** (30.07)	−208.2*** (29.61)
Other Neurological Disorders	−0.0118*** (0.00113)	−0.0118*** (0.00112)	264.9*** (15.24)	253.1*** (14.95)
Chronic Pulmonary Disease	0.0732*** (0.00128)	0.0735*** (0.00129)	117.0*** (12.97)	127.7*** (12.75)
Diabetes, Uncomplicated	0.00198 (0.00128)	0.00219* (0.00127)	−97.99*** (12.32)	−72.09*** (12.06)
Diabetes, Complicated	0.00225 (0.00177)	0.00186 (0.00178)	67.25*** (19.73)	55.87*** (19.39)
Hypothyroidism	−0.00399*** (0.00111)	− 0.00364*** (0.00111)	−14.52 (13.64)	5.217 (13.41)
Renal Failure	−0.00274 (0.00211)	− 0.00240 (0.00212)	−253.4*** (23.85)	−199.1*** (23.48)
Liver Disease	−0.00112 (0.00198)	−0.000870 (0.00200)	−4.929 (25.74)	52.61** (25.23)
Peptic Ulcer Disease Excluding Bleeding	3.80e-05 (0.00301)	0.000122 (0.00301)	679.1*** (62.99)	683.4*** (62.44)
AIDS/HIV	0.00824 (0.00530)	0.00789 (0.00534)	− 480.7*** (62.79)	− 461.0*** (61.70)
Lymphoma	−0.0122*** (0.00349)	−0.0126*** (0.00352)	−189.0*** (54.04)	− 154.1*** (53.11)
Metastatic Cancer	0.00144 (0.00485)	0.00159 (0.00489)	− 509.1*** (59.03)	− 436.3*** (58.17)
Solid Tumor Without Metastasis	−0.00705*** (0.00211)	− 0.00725*** (0.00212)	− 241.3*** (23.76)	−232.8*** (23.46)
Rheumatoid Arthritis/Collagen Vascular	−0.00850*** (0.00154)	−0.00859*** (0.00154)	92.71*** (23.76)	112.1*** (23.34)
Coagulopathy	−0.00653*** (0.00209)	−0.00644*** (0.00207)	122.6*** (32.15)	113.1*** (31.68)
Obesity	0.0105*** (0.00127)	0.0107*** (0.00127)	107.9*** (15.47)	110.5*** (15.17)
Weight Loss	−0.00629*** (0.00149)	−0.00661*** (0.00150)	−63.30*** (20.03)	−59.96*** (19.78)
Fluid and Electrolyte Disorders	0.00810*** (0.00127)	0.00840*** (0.00127)	562.3*** (14.28)	549.7*** (14.06)
Blood Loss Anemia	− 0.00294 (0.00354)	− 0.00289 (0.00354)	13.07 (59.89)	0.0154 (58.85)
Deficiency Anemia	0.00108 (0.00166)	0.000522 (0.00166)	171.1*** (25.74)	181.0*** (25.37)
Alcohol Abuse	−0.000863 (0.00238)	−0.00112 (0.00240)	65.46** (28.96)	56.10** (28.59)
Drug Abuse	0.0121*** (0.00285)	0.0124*** (0.00285)	1044*** (33.54)	1064*** (33.19)
Psychoses	−0.00443* (0.00229)	− 0.00456** (0.00229)	232.5*** (19.44)	223.4*** (19.29)
Depression	−0.00645*** (0.00107)	−0.00605*** (0.00107)	241.2*** (12.74)	259.4*** (12.54)
Hypertension, Complicated	−0.00166 (0.00166)	− 0.00220 (0.00166)	8.475 (19.42)	−39.21** (19.36)
Constant	0.0119*** (0.00183)	0.0125*** (0.00424)	1897*** (20.67)	1037*** (30.23)
Observations	447,867	447,851	447,867	447,851
R-squared	0.022	0.023	0.061	0.092

Robust standard errors in parentheses

*** *p* < 0.01, ** *p* < 0.05, * *p* < 0.1

**Table 3:** Differences in differences for inpatient admissions

Variables	Avoidable Hospitalization		LOS		Total Charges		SNF placement	
	(1)	(2)	(3)	(4)	(5)	(6)	(7)	(8)
Post	0.0144*** (0.00282)	0.0145*** (0.00282)	− 0.229*** (0.0799)	−0.228*** (0.0799)	3839*** (429.2)	3947*** (425.4)	0.0242*** (0.00242)	0.0238*** (0.00242)
Treatment	−0.0202** (0.00864)	−0.0141 (0.00865)	− 0.862*** (0.242)	− 0.955*** (0.244)	− 2651** (1142)	− 3090*** (1144)	−0.148*** (0.00686)	− 0.151*** (0.00686)
Post X treatment	0.0410** (0.0169)	0.0429** (0.0169)	1.111** (0.464)	1.183** (0.464)	555.1 (2251)	1232 (2233)	0.00161 (0.0132)	−0.00180 (0.0131)
Age	0.00189*** (9.25e-05)	0.00188*** (9.23e-05)	−0.00395 (0.00262)	− 0.00427 (0.00262)	− 288.1*** (13.83)	− 280.9*** (13.77)	0.00999*** (7.39e-05)	0.00996*** (7.39e-05)
Female	−0.000219 (0.00280)	− 0.000956 (0.00280)	− 0.494*** (0.0828)	− 0.511*** (0.0828)	− 5962*** (444.9)	− 6007*** (442.0)	−0.0100*** (0.00239)	− 0.00991*** (0.00238)
Urban	−0.0205*** (0.00285)	−0.00817 (0.00587)	0.321*** (0.0787)	0.166 (0.177)	4842*** (405.4)	1476* (894.4)	0.0170*** (0.00241)	0.00633 (0.00570)
Black	0.0160*** (0.00284)	0.0204*** (0.00296)	0.743*** (0.0841)	0.360*** (0.0875)	−543.1 (436.2)	− 1075** (457.6)	−0.0448*** (0.00251)	− 0.0403*** (0.00263)
Other race	0.0153*** (0.00432)	0.0200*** (0.00433)	0.360*** (0.125)	0.105 (0.126)	− 2025*** (639.8)	− 2436*** (644.3)	− 0.0654*** (0.00324)	− 0.0620*** (0.00328)
CHARLSON INDEX	−0.0186*** (0.00179)	− 0.0186*** (0.00179)	0.142*** (0.0516)	0.131** (0.0517)	492.7* (265.6)	514.4* (263.9)	0.0217*** (0.00159)	0.0220*** (0.00159)
Congestive Heart Failure	0.167*** (0.00403)	0.165*** (0.00404)	0.426*** (0.111)	0.468*** (0.111)	2853*** (568.9)	3003*** (565.1)	− 0.00589* (0.00343)	− 0.00788** (0.00343)
Cardiac Arrhythmias	0.00692** (0.00316)	0.00775** (0.00316)	0.683*** (0.0910)	0.649*** (0.0911)	4010*** (479.1)	4246*** (476.3)	− 0.00259 (0.00264)	− 0.00140 (0.00264)
Valvular Disease	0.00399 (0.00423)	0.00646 (0.00427)	0.246** (0.112)	0.158 (0.113)	7466*** (657.9)	5418*** (658.6)	−0.0273*** (0.00342)	− 0.0210*** (0.00346)
Pulmonary Circulation Disorders	0.00458 (0.00528)	0.00909* (0.00528)	1.088*** (0.136)	1.004*** (0.136)	4817*** (754.0)	5205*** (746.7)	0.0211*** (0.00408)	0.0187*** (0.00408)
Peripheral Vascular Disorders	−0.00981** (0.00459)	− 0.00918** (0.00458)	0.289** (0.127)	0.302** (0.128)	8542*** (716.2)	8862*** (711.6)	−0.0220*** (0.00394)	− 0.0230*** (0.00394)
Hypertension, Uncomplicated	−0.00244 (0.00291)	− 0.00434 (0.00291)	−1.182*** (0.0902)	−1.141*** (0.0903)	− 4543*** (474.4)	− 4725*** (471.5)	−0.0648*** (0.00265)	− 0.0643*** (0.00266)
Paralysis	−0.0115* (0.00693)	− 0.0124* (0.00690)	1.758*** (0.246)	1.785*** (0.246)	6502*** (1242)	6439*** (1235)	0.0551*** (0.00690)	0.0553*** (0.00689)
Other Neurological Disorders	−0.0377*** (0.00330)	−0.0340*** (0.00329)	1.101*** (0.103)	1.084*** (0.104)	4892*** (529.1)	4664*** (527.5)	0.112*** (0.00313)	0.109*** (0.00313)
Chronic Pulmonary Disease	0.138*** (0.00340)	0.136*** (0.00340)	−0.698*** (0.0948)	−0.606*** (0.0949)	− 852.2* (497.6)	− 677.7 (494.4)	− 0.0827*** (0.00286)	− 0.0834*** (0.00286)
Diabetes, Uncomplicated	0.0653*** (0.00347)	0.0656*** (0.00347)	−0.252** (0.101)	−0.249** (0.101)	1735*** (521.1)	1165** (517.7)	− 0.0182*** (0.00309)	− 0.0183*** (0.00309)
Diabetes, Complicated	0.162*** (0.00514)	0.161*** (0.00513)	0.520*** (0.138)	0.495*** (0.138)	− 1026 (722.3)	−550.3 (717.1)	− 0.0155*** (0.00430)	− 0.0155*** (0.00429)
Hypothyroidism	−0.0167*** (0.00386)	− 0.0167*** (0.00385)	− 0.188* (0.106)	− 0.142 (0.106)	− 1470*** (536.4)	− 1625*** (531.5)	0.000919 (0.00335)	0.000201 (0.00334)
Renal Failure	0.0993*** (0.00628)	0.0979*** (0.00627)	0.744*** (0.173)	0.828*** (0.173)	5868*** (954.6)	5843*** (949.4)	−0.0295*** (0.00537)	− 0.0297*** (0.00537)
Liver Disease	−0.00479 (0.00582)	−0.00413 (0.00581)	0.0679 (0.176)	0.171 (0.177)	1089 (946.3)	2005** (943.8)	− 0.0634*** (0.00441)	−0.0653*** (0.00442)
Peptic Ulcer Disease Excluding Bleeding	−0.0223*** (0.00845)	− 0.0197** (0.00844)	0.350 (0.259)	0.361 (0.259)	2312 (1616)	2188 (1611)	−0.0489*** (0.00700)	− 0.0511*** (0.00698)
AIDS/HIV	0.0467*** (0.0150)	0.0495*** (0.0150)	−0.344 (0.485)	− 0.421 (0.485)	1731 (2776)	1425 (2761)	− 0.132*** (0.0122)	− 0.132*** (0.0122)
Lymphoma	0.0191 (0.0126)	0.0225* (0.0126)	1.472*** (0.373)	1.512*** (0.372)	9788*** (2084)	10,119*** (2074)	−0.0813*** (0.0101)	−0.0810*** (0.0100)
Metastatic Cancer	0.0446*** (0.0131)	0.0457*** (0.0131)	−0.519 (0.398)	−0.492 (0.399)	− 3457* (2065)	− 3342 (2049)	−0.177*** (0.0116)	− 0.178*** (0.0116)
Solid Tumor Without Metastasis	0.00197 (0.00618)	0.00247 (0.00618)	0.101 (0.178)	0.104 (0.178)	4479*** (961.6)	4363*** (952.8)	−0.103*** (0.00516)	−0.104*** (0.00516)
Rheumatoid Arthritis/Collagen Vascular	−0.0297*** (0.00545)	−0.0287*** (0.00545)	− 0.397*** (0.151)	−0.374** (0.151)	673.3 (829.7)	806.4 (824.9)	−0.0225*** (0.00441)	−0.0204*** (0.00441)
Coagulopathy	−0.0331*** (0.00515)	− 0.0349*** (0.00514)	0.782*** (0.156)	0.730*** (0.156)	6034*** (830.9)	6450*** (826.5)	−0.00367 (0.00438)	− 0.00422 (0.00438)
Obesity	−0.0112*** (0.00390)	−0.0112*** (0.00390)	− 0.445*** (0.100)	−0.455*** (0.100)	− 869.6 (553.6)	90.22 (550.6)	− 0.00462 (0.00285)	− 0.00625** (0.00285)
Weight Loss	0.00249 (0.00425)	0.00491 (0.00425)	2.612*** (0.136)	2.569*** (0.136)	13,957*** (733.5)	14,321*** (728.8)	0.0763*** (0.00392)	0.0773*** (0.00392)
Fluid and Electrolyte Disorders	0.0684*** (0.00285)	0.0649*** (0.00286)	0.309*** (0.0809)	0.357*** (0.0810)	−491.2 (425.9)	−690.2 (423.4)	0.0142*** (0.00241)	0.0149*** (0.00241)
Blood Loss Anemia	−0.0704*** (0.00933)	−0.0741*** (0.00932)	0.448* (0.263)	0.545** (0.262)	4827*** (1443)	4920*** (1425)	0.00792 (0.00828)	0.00764 (0.00826)
Deficiency Anemia	0.00181 (0.00465)	0.00234 (0.00463)	0.263** (0.129)	0.237* (0.129)	− 737.3 (659.8)	−22.50 (655.8)	0.0101*** (0.00373)	0.00913** (0.00373)
Alcohol Abuse	−0.0465*** (0.00541)	−0.0457*** (0.00540)	−0.533*** (0.170)	−0.576*** (0.170)	− 5492*** (852.2)	− 5410*** (848.0)	−0.0446*** (0.00403)	− 0.0440*** (0.00403)
Drug Abuse	0.0249*** (0.00560)	0.0258*** (0.00559)	0.101 (0.156)	0.126 (0.156)	− 4140*** (771.4)	− 3867*** (765.0)	−0.0451*** (0.00318)	−0.0440*** (0.00318)
Psychoses	−0.0310*** (0.00455)	− 0.0299*** (0.00455)	2.708*** (0.167)	2.616*** (0.167)	− 4081*** (674.3)	− 3745*** (672.4)	0.0630*** (0.00418)	0.0619*** (0.00418)
Depression	−0.00661** (0.00328)	−0.00401 (0.00328)	− 0.307*** (0.0923)	−0.274*** (0.0928)	− 3703*** (479.4)	− 3587*** (478.0)	0.0330*** (0.00270)	0.0304*** (0.00271)
Hypertension, Complicated	0.00145 (0.00536)	0.00527 (0.00536)	−0.348** (0.141)	−0.414*** (0.142)	− 3307*** (793.5)	− 3350*** (789.7)	−0.0227*** (0.00447)	− 0.0230*** (0.00447)
Constant	0.0885*** (0.00658)	0.0355** (0.0163)	8.463*** (0.194)	8.215*** (0.574)	76,682*** (1067)	66,435*** (2507)	−0.323*** (0.00504)	−0.364*** (0.0141)
Observations	199,649	199,643	199,649	199,643	199,649	199,643	199,649	199,643
R-squared	0.073	0.078	0.015	0.018	0.019	0.036	0.123	0.127

Robust standard errors in parentheses

*** *p* < 0.01, ** *p* < 0.05, * *p* < 0.1

## Discussion

Healthy Connections Prime (HCP) was expected to move more than 53,000 South Carolina seniors eligible for both Medicare and Medicaid coverage into integrated managed care programs [31]. But the results as of 2016 show that dual-eligibles in South Carolina were very hesitant to join HCP; and that for those who do, do not show statistically significant improvements in various health outcomes. Dual-eligibles with certain comorbidities (hypertension and vascular disease, COPD, diabetes, renal failure, liver disease, AIDS and cancer) and higher Charlson comorbidity indices are less likely to sign up for HCP. Once enrolled, patients with more complex conditions appear more likely to drop out.

South Carolina’s low enrollment is similar to other states that are in various stages of demonstration project development, whose initial enrollments generally ranged from 5.3 to 29% [24]. Only three states exceeded 30% in enrollment, including Michigan (35.8%), Virginia (42.5%), and Ohio (62.4%). These early findings call into question whether passive enrollment will necessarily encourage dual-eligibles to participate in integrated care models. South Carolina, like 10 of the 13 demonstration states, chose an enrollment process in which an initial period of voluntary enrollment is followed by passive enrollment. Therefore, our findings are particularly surprising because in South Carolina, enrollment in HCP is the default option for all dually eligible individuals, and occurs unless a Medicare-Medicaid dual eligible enrollee takes the extra effort to opt out of HCP. Indeed, existing literature in behavioral economics shows that individuals often passively “choose” the default presented to them because faced with complex decisions, simple heuristics and rules of thumb are easier to follow than elaborate problem-solving [32–37].

Besides low rates of initial enrollment, another problem facing HCP is patients with multi-morbidity are more likely to opt out of the program. We did not interview patients to assess their reason for disenrolling from a program that is intended to improve their care. However, the Massachusetts study found that patients feared losing relationships with trusted providers [38]. They also were worried they might face new service restrictions. It is possible that had HCP done more education outreach early on with this population, especially with providers, the dropout rates would be lower. The elderly poor are likely a difficult group to target efficiently for education and outreach. Nevertheless, almost all states that chose the capitated model^4^ for their demonstration project contract with a third party that markets and promotes the integrated program to dually eligible populations subject to stringent state laws on marketing. This critical period can be used to provide information that is tailored to the concerns of dual eligibles who choose to opt out of or later disenroll from HCP or equivalent programs in other states.

Our findings in health outcomes differ from those in the Washington State’s preliminary evaluation [39]. The reported preliminary outcome measures indicated that 30-day all-cause risk-standardized hospital readmission rates dropped from 22.9 to 17.7% during the baseline period. During the demonstration period, the readmission rate continued to drop to 15.4%. ED visits per 1000 eligibles dropped from 200.5 to 184.9 during the baseline period. During the demonstration period, it increased to 189.2. Evaluations of these measures should be interpreted with caution, however, as they have not been tested for significance and a conclusive connection cannot be made between the measure outcomes and the related to the demonstration. In addition, due to relatively short post-period, our findings may not capture HCPs true effects on health outcomes.

Further study should also focus on the design of the integrated payment system, as it is possible that participating physicians are concerned about protecting their revenue stream in a system designed to reduce redundant care. Beyond quantitative studies of secondary data, future work should include qualitative studies that evaluate how HCP is being implemented in the field and in practice. Many questions remain to be resolved, such as: Did clinical integration actually result in better coordination among health professionals to provide integrated care? What level of understanding do patients have about the potential benefits of integrating the two systems, in terms of quality of care, better experiences and improved health outcomes? Understanding the barriers on both the patient demand and provider supply side will be instrumental in improving the enrollment process for HCP and other demonstration projects. Above all, it is essential that further studies be conducted to assess whether the Financial Alignment Initiative improves health outcomes, as theory predicts. If so, a question is whether these demonstrative projects should implement mandatory enrollment, which would entail legal challenges because by law Medicare must provide enrollees a choice between a managed care and fee-for-service program. Other methods to encourage enrollment may be required.

This research has several limitations. The Emergency Department Algorithm (EDA) developed at New York University uses administrative discharge data to distill hundreds of International Classification of Diseases-9 codes for emergency department (ED) visits into 4 categories. Therefore, the Algorithm may be insufficiently sensitive to changes in ED utilization patterns to be useful in assessing HCP’s effects. Also, despite the unique strengths of the AHRQ PQIs, for some PQIs, differences in socioeconomic status have been shown to explain a substantial part of the variation in PQI rates across areas. To address these concerns, we also conducted all-cause ED and inpatient admissions, and do not find significant differences in results. Moreover, for all regressions, we also controlled for observable measures of socioeconomic status such as race and ethnicity to attenuate the concern that socioeconomic status drove any results related to PQIs.

Although the results of this early evaluation do not indicate HCP achieved its intended goals, and may cause policymakers to question the success of the demonstration, experience from states such as Minnesota and Massachusetts—which had a fifteen-year history of integrating Medicare and Medicaid services for dual-eligible beneficiaries aged 65 and over—indicates that care integration may lead to improved outcomes [38, 40]. Positive outcomes include a reduction in ED visits and hospital admissions. Moreover, given that so few dual eligibles enrolled in the earlier waves with longer follow-up periods, positive outcomes may require time to manifest themselves. Further observation and more structural changes in the demonstrations, such as better alignment of program administration, greater account of provider incentives, and greater up-front infrastructure investments in the early years, could potentially result in long-term savings, improved quality of care, and greater availability of services in the home, rather than costlier institutional care. We hope that the observed early challenges may be remedied with revisions in law and program guidelines.

## Conclusion

Although HCP was implemented with the goal of improving the coordination of Medicare and Medicaid services for dual eligible in South Carolina, our results show that uptake of HCP was very low. People with multi-morbidity and greater severity of illness are less likely to join HCP, and once they join, are more likely to quit. In addition, our analyses show that there was little difference in observable measures of health outcomes, including avoidable ED admissions and avoidable inpatient admissions, between HCP and non-HCP members. The early results from this study show that HCP may require further refinement to encourage enrollment and achieve its stated purpose of reducing avoidable ED and inpatient admissions.

## Abbreviations

AHRQ PQI: Agency for Healthcare Research and Quality Prevention Quality Indicators
AIDS: Acquired immune deficiency syndrome
CICO: Coordinated and integrated care organizations
CMS: Centers for medicare and medicaid services
COPD: Chronic obstructive pulmonary disease
DID: Difference-in-differences
ED: Emergency department
EDA: Emergency Department Algorithm
HCP: Healthy Connections Prime (the Medicare-Medicaid Financial Alignment Demonstration Project in South Carolina)
LOS: Length of stay
RFA: Revenue and Fiscal Affairs Office (of South Carolina)
SCO: Senior care option

## Footnotes

1 Individuals under 65 may also qualify for Medicare if they receive Social Security Disability Insurance, or if they are diagnosed with End Stage Renal Disease. As of 2016, 84.5% of all Medicare beneficiaries are 65 and older. (See https://www.statista.com/statistics/248035/distribution-of-medicare-beneficiaries-by-age/)

2 Medicaid is a joint federal and state-funded insurance program, and eligibility requirements differ by state. In all states, Medicaid covers some low-income individuals, families and children, pregnant women, the elderly, and those with disabilities. In some states the program covers all low-income adults below a certain income level.

3 Dual eligibles lose their eligibility primarily by earning too much income to qualify for Medicaid.

4 In the capitated model, CMS and the state will pay a predetermined set amount to the health plan that administers the financial alignment demonstration project in the state.
